# Inhibitory control in the sober state as a function of alcohol sensitivity: a pilot functional magnetic resonance imaging (fMRI) study

**DOI:** 10.3389/fnhum.2025.1557661

**Published:** 2025-02-28

**Authors:** Roberto U. Cofresí, Spencer Upton, Devon Terry, Alexander A. Brown, Thomas M. Piasecki, Bruce D. Bartholow, Brett Froeliger

**Affiliations:** ^1^Department of Psychological Sciences, University of Missouri, Columbia, MO, United States; ^2^Department of Medicine, Center for Tobacco Research and Intervention, University of Wisconsin, Madison, WI, United States; ^3^Department of Psychological and Brain Sciences, University of Iowa, Iowa City, IA, United States; ^4^Department of Psychiatry, University of Missouri, Columbia, MO, United States

**Keywords:** craving, executive function, cingulate, cognitive control, response inhibition

## Abstract

**Introduction:**

Lower sensitivity (LS) to acute alcohol promotes hazardous alcohol use, increasing risk for alcohol use disorder (AUD). Compared to peers with high sensitivity (HS), LS individuals exhibit amplified responses to alcohol cues and difficulty exerting inhibitory control (IC) over those cued responses. However, it is unclear whether LS and HS individuals differ in neural or behavioral responses when exerting IC over affectively neutral prepotent responses (i.e., domain-general IC). This fMRI pilot study examined domain-general IC and its neural correlates in young adult LS and HS individuals.

**Methods:**

Participants (*N* = 32, M_*age*_ = 20.3) were recruited based on their Alcohol Sensitivity Questionnaire responses (HS: *n* = 16; LS: *n* = 16; 9 females/group) to complete an event-related fMRI IC task in a sober state. Retrospective assessments of alcohol craving, consumption, and problems were taken outside the lab.

**Results:**

Although IC performance (accuracy) was numerically lower for the LS group (M[SD] = 0.527[0.125]) compared to the HS group (M[SD] = 0.595[0.124]), no significant difference was detected [*t*(30) = 1.55, *p* = 0.132]. Across groups, IC-related activity was observed in bilateral fronto-cortico-striatal circuitry, including dorsal striatum (DS) and dorsal/supragenual anterior cingulate cortex (dACC). Within group HS, IC-related dACC activity was greater among individuals reporting less intense (b-95 CI = [−0.201, −0.041], *p* = 0.004) and less frequent alcohol craving experiences (b-95 CI = [−0.131, 0.005], *p* = 0.068), whereas in group LS, IC-related dACC activity was greater among individuals reporting more intense (b-95 CI = [0.009, 0.140], *p* = 0.028) and more frequent alcohol craving experiences (b-95 CI = [0.022, 0.128], *p* = 0.007).

**Discussion:**

In sum, while LS and HS individuals demonstrated similar domain-general IC performance and recruited similar neural resources to perform IC, findings suggest that compensatory over-activation of frontocortical nodes of the fronto-cortico-striatal IC circuitry may be related to affective-motivational aspects of AUD symptomatology (craving in daily life) among LS individuals. Based on these preliminary findings, future studies with larger samples are warranted to determine the extent to which domain-general IC performance associated with fronto-cortico-striatal IC circuit activation contributes to the alcohol use pathophysiology, and whether therapeutic interventions (e.g., non-invasive brain stimulation) targeting fronto-cortico-striatal IC circuitry may decrease AUD symptomatology.

## 1 Introduction

Alcohol use is highly prevalent and socially acceptable compared to the use of other addictive substances, yet poses a myriad of acute and chronic harms to individuals and society (Connor, 2017; Duke et al., 2017; Griswold et al., 2018). Chronic use of alcohol, like other addictive substances, is associated with deficits in multiple forms of executive functioning (EF) (Brion et al., 2017; Fernández-Serrano et al., 2011). Individual differences in inhibitory control (IC), one facet of EF, are strongly implicated in risk for onset and relapse across various substance use disorders (SUDs), including alcohol use disorders (AUD) (Gunawan et al., 2024; Morein-Zamir and Robbins, 2015; Smith et al., 2014; Zilverstand et al., 2018). IC encompasses the cognitive ability to stop a prepotent response (Friedman and Miyake, 2004, 2017; Miyake et al., 2000). Lower IC task performance is associated with heavier and more hazardous patterns of alcohol use (Hu et al., 2016; López-Caneda et al., 2014; Mullan et al., 2011; Murphy and Garavan, 2011; O’Halloran et al., 2020) and higher likelihood of relapse (return to use) among individuals attempting to abstain from drinking alcohol (Czapla et al., 2016; Rupp et al., 2016). The associations between IC and AUD risk may be driven by individual differences in susceptibility to the acute effects of alcohol, including IC impairment, as well as by individual differences in IC abilities in the sober state.

Implementation of IC involves at least one of two potentially cooperative frontocortical-basal ganglia circuits (Bundt and Huster, 2024; Hannah and Aron, 2021; Jahanshahi et al., 2015; Wessel and Anderson, 2024): the indirect and hyperdirect pathways. The indirect IC pathway involves frontocortical excitatory drive onto cells in dorsal striatum (caudate/putamen) with inhibitory projections onto cells in globus pallidus pars externa (GPe) that in turn have inhibitory projections onto cells in globus pallidus pars interna (GPi) and substantia nigra pars reticulata (SNr) that provide inhibitory tone on thalamic cells with excitatory projections to the primary motor cortex (M1) in the precentral gyrus. The hyperdirect IC pathway involves frontocortical excitatory drive onto cells in the subthalamic nucleus (STN) with excitatory projections onto the inhibitory cells in GPi/SNr, providing a faster mechanism (in terms of fewer synapses) for inhibition of M1. Meta-analyses suggest that key frontocortical nodes for IC implementation include dorsal and ventral lateral frontal cortices, such as anterior insula, middle and inferior frontal gyrus, as well as more medial cortices, such as anterior cingulate and superior frontal gyrus, depending on task-specific demands (Criaud and Boulinguez, 2013; Gavazzi et al., 2021, 2023; Isherwood et al., 2021; Simmonds et al., 2008; Zhang et al., 2017).

Individuals with active SUDs tend to exhibit hypo-activation of frontocortical IC circuit nodes during successful IC compared to healthy control cases (Goldstein and Volkow, 2002; Le et al., 2021; Luijten et al., 2014; Moeller et al., 2016; Zilverstand et al., 2018), suggesting that, functionally, “under-recruitment” of IC circuits lies at the core of SUD-related IC deficits. However, the story is complicated by potential differences between SUDs as well as by differential relationships to different aspects of risk (e.g., craving, consumption, and consequences) across the lifespan and/or substance use trajectory (Heitzeg et al., 2015; Hildebrandt et al., 2021; Luijten et al., 2014; Moeller et al., 2016). Indeed, as noted by Moeller et al. (2016), hyper- rather than hypo-activation of frontocortical IC circuit nodes during successful IC has been associated with craving and relapse (return to use) risk in clinical samples (Froeliger et al., 2017; Grieder et al., 2022; Prisciandaro et al., 2013; Stein et al., 2021), as well as with risky substance use patterns (e.g., binge drinking) in non-clinical samples (Herman et al., 2019; Suárez-Suárez et al., 2020). To reconcile such findings with the over-arching idea that SUD-related IC deficits are due to “under-recruitment” of IC circuits, it has been proposed that to achieve certain levels of IC performance some individuals compensate for hypo-active IC circuits by expending additional effort or neural resources (Heitzeg et al., 2015; Hildebrandt et al., 2021), leading to apparent “over-recruitment” of IC circuits.

### 1.1 Low sensitivity to alcohol: an endophenotype of AUD risk

Individual differences in susceptibility to alcohol intoxication are known to moderate risk for AUD onset and progression. Specifically, lower sensitivity (LS) to acute alcohol predicts heavier alcohol use and more alcohol use-related problems, including AUD (Schuckit et al., 2007, 2017, 2021). Several mechanisms have been proposed to account for LS-related AUD risk, including paradoxical hyper-sensitivity to appetitive effects of alcohol (Fleming et al., 2016; King et al., 2021, 2022), including cue reactivity (Bartholow et al., 2010; Cofresí et al., 2022b), and hypo-sensitivity to aversive effects (Davis et al., 2021; Fleming et al., 2016; Hone et al., 2017; Piasecki et al., 2012). Despite continued empirical and theoretical progress in understanding LS-related AUD risk (Parker et al., 2020; Ray et al., 2010; Schuckit et al., 2021), the role of potential sober-state differences in EF facets like IC in LS-related AUD risk remains under-explored.

Early indication of potential sober-state differences in EF-related processes as a function of alcohol sensitivity phenotype came from an attentional IC (flanker task) study using event-related brain potentials. This study found that, despite similar task performance, the P300 was smaller among LS individuals relative to HS peers (Bartholow et al., 2003). The P300 is an attention-related brain potential that integrates activity across multiple distributed neural systems (Linden, 2005; Polich, 2007). To our knowledge, there has been only one prior fMRI study of IC performance as a function of alcohol sensitivity phenotype, that is, only one prior study that would be able to identify the specific neural substrates of potential sober-state differences in IC processes. This study found that, at matched IC performance levels, sober LS individuals “over-recruit” anterior (frontal, cingulate, precentral) cortical areas during successful IC compared to their HS peers (Schuckit et al., 2012). Consequently, LS and HS individuals may or may not differ in IC abilities *per se*, yet may differ in terms of frontocortical circuitry recruitment to successfully implement IC.

Replicating the latter finding and establishing the extent to which IC performance and its neural substrates differ between LS and HS individuals is important for understanding the potential role of domain-general (viz., “core”) self-control abilities in shaping LS and HS individuals’ different alcohol craving and consumption topographies in the natural environment (Kohen et al., 2023; Piasecki et al., 2012; Trela et al., 2016, 2018). Furthermore, potential differences in the neural substrates of IC as a function of alcohol sensitivity phenotype can help inform early-stage indicators for risk of developing AUD, as well as provide guidance on neuroanatomical locations for testing non-invasive brain stimulation (NIBS) to modulate IC in the context of AUD.

### 1.2 The current study

A functional magnetic resonance imaging (fMRI) pilot study was conducted to examine potential differences in sober-state domain-general IC performance and its neural underpinnings among young adults who regularly use alcohol and report relatively extreme LS or HS to acute alcohol. Based on (Schuckit et al., 2012), it was hypothesized that IC performance would be similar between groups, but that successful IC performance would be associated with elevated activity, as indexed by the blood oxygen-level dependent (BOLD) response, in the frontocortical nodes of the IC circuits for group LS compared to HS. Given an extensive literature linking LS to alcohol with elevated alcohol craving, consumption, and consequences, alcohol sensitivity phenotype-based effects on IC circuit activation were examined while accounting for potential moderation by between-person differences in alcohol use and problem levels or daily experiences with alcohol craving.

## 2 Materials and methods

This report presents primary analysis of behavioral task performance and brain activity measures derived from a domain-general IC task completed in the context of a functional neuroimaging pilot study focused on alcohol cue reactivity among young adults with relatively extreme LS or HS to acute alcohol. Detailed description of the sample, including its sociodemographics and alcohol use behavior, as well as in-depth coverage of laboratory visit procedures were published in our recent report on the alcohol cue reactivity results of the study (Cofresí et al., 2024). Below, we provide brief coverage where details are available in our recent report, and in-depth coverage of aspects relevant to the IC task and its analysis.

### 2.1 Participants

Participants for the fMRI pilot study were recruited from a 95 pool of individuals who were actively involved in or had recently (past year) completed a NIH-funded longitudinal study (AA025451) characterizing alcohol sensitivity across early emerging adulthood. Eligibility criteria for the longitudinal (“parent”) study have been previously reported (Cofresí et al., 2022a; Cofresí et al., 2022b; Kohen et al., 2023, 2024). Briefly, inclusion criteria at time of enrollment in the parent study included: (1) being age 18–20; (2) English language proficiency; (3) normal or corrected-to-normal vision; and (4) regular alcohol use (at least monthly use across past year, and at least 1 binge drinking episode in past 6 months). Exclusion criteria at time of enrollment in the parent study included: (1) a history of unsuccessful attempts to moderate or quit alcohol use; (2) any current or past psychosis; (4) any history of major chronic illness or neurological disease (e.g., epilepsy); (5) any history of head injury that resulted in loss of consciousness; or any (6) EEG contraindications (e.g., highly sensitive skin, hairstyle preventing scalp access for electrode placements). Individuals were excluded from potential participation in the fMRI pilot study if they reported any of the following at subsequent screening: (1) no longer living in or near Columbia, MO or inability or unwillingness to travel to the lab again for a new study; (2) MRI contraindications (e.g., claustrophobia, non-removable medical electronics or ferrous metal in the body, sensitivity to loud noises); (3) new history of unsuccessful attempts to moderate or quit alcohol use; (4) irregular (less than monthly) or no alcohol use in past year; (5) any current or new history past psychosis; (6) new history of major chronic illness or neurological disease; (5) new history of head injury that resulted in loss of consciousness. From the remaining pool of 76 otherwise eligible individuals, 15 (7 females, 8 males) were excluded from potential participation due to moderate alcohol sensitivity phenotype (see “2.3.1. Alcohol Sensitivity Questionnaire (ASQ)” for details). This left 61 prospective participants, only 36 of whom could possibly be enrolled due to the limited funds available for the fMRI pilot study. Prospective participants were contacted and enrolled strategically to ensure similarly sized HS and LS groups, and similar numbers of females and males within these groups. Of 34 individuals scheduled for participation prior to the end of the enrollment (and funding) period, only 33 visited the lab (1 person failed to present themselves and did not reply to rescheduling attempts). Additional exclusion criteria at the time of the MRI scan were: (1) alcohol intoxication; and for female participants: (2) pregnancy, trying to become pregnant, and/or breastfeeding. One individual who visited the lab to participate in the fMRI pilot study was able to tolerate procedures in the training/mock scanner, but unable to do so in the actual MRI scanner due to unexpected claustrophobia—this person’s data were excluded from all analyses. Ultimately, 32 participants (age 18–23 years at time of MRI scans; 56% female, 94% Non-Hispanic White, 91% Right-Handed), 16 HS and 16 LS, were included in the final analytic sample for this study. For more sociodemographic details, see our recent report also based on this sample (Cofresí et al., 2024).

### 2.2 Procedures

All procedures were approved by the University of Missouri Institutional Review Board. Laboratory visits lasted ∼2 h and took place at the University of Missouri Cognitive Neuroscience Systems Core facility. Sobriety was verified with a breath alcohol test upon arrival and then informed consent was obtained. To rule out claustrophobia in the MRI environment, participants underwent training in a mock MRI scanner. Urine samples were collected and tested for cotinine (Healgen One Step COT, Healgen Scientific LLC, Houston, TX, USA), and for female participants, pregnancy (Sure-Vue hCG-STAT, Fisher Healthcare, Pittsburgh, PA, USA). All participants then underwent the MRI phase of the study, which included a ∼7-min IC task (see “2.4.3. IC fMRI task” for details) and a ∼30-min cue reactivity task (previously reported: Cofresí et al., 2024). Finally, outside of the scanner, participants completed an 8-day TimeLine Follow-Back calendar (Sobell and Sobell, 1992), were debriefed, and compensated ($50 USD).

### 2.3 Questionnaires

Alcohol sensitivity and AUD risk levels were assessed before the lab visit using electronically administered surveys (Harris et al., 2009), whereas recent alcohol craving experiences were assessed at the lab visit between the mock MRI scanner training and the MRI phase of the study. Assessments were conducted using validated, standardized questionnaire instruments (described next). For more details about alcohol and other substance use in this sample, see our recent report (Cofresí et al., 2024).

#### 2.3.1 Alcohol Sensitivity Questionnaire (ASQ)

Each participant’s general sensitivity to the acute effects of alcohol was assessed using the ASQ (Fleming et al., 2016). The ASQ’s 15 items each query whether the respondent has experienced a specific effect from drinking alcohol (e.g., feeling buzzed; passing out), and for all endorsed effects respondents indicate the number of standard drinks they typically require to experience it. For current purposes, responses (i.e., numbers of drinks) across all 15 items were averaged to produce the ASQ total score. These scores were used to classify participants as either LS or HS based on upper and lower terciles of the sex-stratified ASQ score distribution. More details about the sex-stratified thresholds are available in Cofresí et al. (2024). Internal consistency reliability (ICR) for ASQ scores was excellent (α = 0.91–0.95). Group LS comprised females with ASQ total scores > 4.50, and males with ASQ total scores > 5.50. Group HS comprised females with ASQ total scores < 3.00, and males with ASQ total scores < 4.50.

#### 2.3.2. Alcohol use disorder identification test (AUDIT)

Past year alcohol use and alcohol-related problem levels were assessed using the AUDIT (Bohn et al., 1995; Saunders et al., 1993) subscales for Consumption and Problems (Peng et al., 2012). Use levels were indexed by summing responses to AUDIT items 1–3 into AUDIT Consumption subscale scores. Problem levels were indexed by summing responses to AUDIT items 4–10 into AUDIT Problem subscale scores. ICR for these AUDIT scores was fair-to-good (α = 0.78–0.88). Supplementary Table 1 shows AUDIT subscale scores were elevated in group LS compared to HS, as previously reported (Cofresí et al., 2024).

#### 2.3.3 Alcohol Craving Experience Questionnaire (ACEQ)

The frequency and strength of alcohol craving in the past week were assessed using subscales of the Frequency and Strength forms of the ACEQ (May et al., 2014; Statham et al., 2011). The Frequency form focused on the frequency of craving experiences (weak or strong) during the past week. The Strength form focused on the strength of the most intense craving experience within the past week by instructing participants to think about the time in the past week when they “most wanted” to use alcohol and to refer to that experience when responding. Craving frequency levels were indexed by summing responses to 3 items (stem: “how often did you…”; items: “want it?”, “need it?,” “have a strong urge for it?”) on the Frequency form into ACEQ-Frequency subscale scores. Peak craving intensity or strength levels were indexed by summing responses to 3 items (stem: “At that time…”; items: “how much did you want it?,” “how much did you need it?,” “how strong was the urge to have it?”) on the Strength form into ACEQ-Strength subscale scores. ICR for these ACEQ subscale scores was good (α = 0.82–0.83). Supplementary Table 1 shows ACEQ subscale scores were elevated in group LS compared to HS, as previously reported (Cofresí et al., 2024).

### 2.4 MRI

#### 2.4.1 Image acquisition

MRI scans were acquired using a 3T Siemens Prisma scanner using a 32-channel head coil with padding to restrict head movements. A high-resolution, T1-weighted magnetization prepared-rapid gradient echo (MPRAGE) sequence (TR = 2,300 ms, TE = 2.26 ms, flip angle = 9°, 192 slices, 1-mm isotropic voxels, FOV = 256 mm) was used to acquire anatomical images. Following the acquisition of a B0 field map, functional T2*-weighted images were acquired to measure BOLD responses using a simultaneous multi-slice (SMS) echo-planar imaging (EPI) sequence (acceleration factor = 3, TR = 2,000 ms, TE = 36 ms, flip angle = 70°, 69 slices, 2.2-mm isotropic voxels, FOV = 207 mm).

#### 2.4.2 Image processing

Functional and structural images underwent standard preprocessing using statistical parametric mapping (SPM) package version 12 (Penny et al., 2007) in Matlab version 2021b (The Mathworks Inc., Natick, MA, USA). Preprocessing included: B0 correction; realignment; slice timing correction; co-registration to structural images; segmentation of structural images; normalization to MNI space using forward deformations with resampling to 1.5-mm^3^ voxels; and smoothing with a 6-mm^3^ full-width at half maximum (FWHM) Gaussian filter.

#### 2.4.3 IC fMRI task

IC was assessed with the “Go/Go/NoGo” task (Chikazoe et al., 2009), which has been validated in SUD populations (Bell and Froeliger, 2021; Brown A. A. et al., 2023; Froeliger et al., 2017; Newman-Norlund et al., 2020; Upton et al., 2023a; Upton et al., 2023b). Using handheld response pads, participants were instructed to press a button in response to common (gray circles: 75.8% of trials) and rare (yellow circles: 12.1% of trials) *Go* stimuli and to inhibit responding to rare *NoGo* stimuli (blue circles: 12.1% of trials). The task provided errors of omission and reaction times during Go trials, errors of commission on NoGo trials (blue circles) and controlled for novelty detection via Rare Go trials (yellow circles). Total task time was 7 min (538 trials, 400 ms stimulus, 400 ms blank); completed in 1 run.

### 2.5 Analytic approach

#### 2.5.1 IC fMRI task—Behavior

IC performance was indexed by adjusted NoGo trial accuracy. As in Bell and Froeliger (2021), Froeliger et al. (2017), Newman-Norlund et al. (2020),and Upton et al. (2023a),b, NoGo trial accuracy was adjusted to control for transient attentional lapses unrelated to IC by scoring NoGo trials with null response as incorrect when the participant did not respond to the Go trial immediately preceding it. IC performance and other task-derived behavioral performance measures [e.g., Go or Rare Go correct response time (RT)] were examined for group differences using two-tailed independent samples Student’s *t*-tests and Wilcoxon rank sum tests, as appropriate.

#### 2.5.2 IC fMRI task—Brain

##### 2.5.2.1 First-level analyses

Preprocessed functional images were entered into a 1st-level analysis using the general linear model (GLM) to examine the BOLD response during each of 5 event types: NoGo_*correct*_ (successful IC), NoGo_*incorrect*_ (error of commission), RareGo_*correct*_ (novel-target detection), RareGo_*incorrect*_ (novel-target error of omission), and Go_*incorrect*_ (error of omission). Each event was modeled as an impulse at event onset (event duration = 0 s) and convolved with a canonical hemodynamic response function. If a person’s data were missing for a given event type (e.g., NoGo_*incorrect*_ because the person always correctly omitted responses to the NoGo stimulus), the specific event type was not included in the 1st-level model of that person’s data. Intra-run motion was removed through rigid body rotation and translation, and 6 motion parameters (x, y, z, roll, pitch, yaw) were included as nuisance covariates. A high-pass filter (128 s; 0.008 Hz) was applied to remove slow signal drift. A whole brain mask was applied. To isolate brain activity during successful IC while controlling for novelty detection, a NoGo_*correct*_–RareGo_*correct*_ contrast image (IC contrast) was generated and fed-forward to 2nd level analyses.

##### 2.5.2.2 Second-level analyses

A whole-brain 2nd level model was fit to the 1st level model-derived IC contrast images to identify voxel clusters showing significant IC-related activity across the full sample (ignoring alcohol sensitivity phenotype groups). The voxel intensity-based statistical threshold was set to family-wise error (FWE)-corrected *p* < 0.05 using the random field theory (RFT) method in SPM, which accounts for image smoothing and the statistical dependency of signal from neighboring voxels. Furthermore, the spatial extent-based statistical (cluster-forming) threshold was set to kE ≥ 3 voxels to avoid detecting intensely activated but spatially isolated voxels since these are more likely to be false positives. Person-level IC contrast beta coefficients were then extracted using MarsBaR version 0.45 (Brett et al., 2002). The average IC contrast beta coefficient in a 5 mm radius sphere centered on the peak voxels in each cluster was extracted. Peak voxel locations are reported using the Montreal Neurological Institute (MNI) coordinate system.

##### 2.5.2.3 Alcohol sensitivity phenotype group-difference hypothesis tests

The predicted group difference (LS > HS) in IC-related neural activity was tested at all extracted functional ROIs using a multiple linear regression (MLR) approach. This approach enables testing and controlling for moderating effects of sex and previously reported group differences (LS > HS) in alcohol use, problems, and cravings (see Supplementary Table 1). Between-person differences in alcohol use (AUDIT Consumption), problems (AUDIT Problem), and cravings (frequency: ACEQ-F; strength: ACEQ-S) were thus tested as moderators of the predicted group difference (LS > HS) in IC-related neural activity. Testing these moderators in separate models was necessary to mitigate collinearity issues arising from large intercorrelations (see Supplementary Table 2). They were entered into the MLR models as grand mean-centered continuous variables. Sex also was tested as a potential moderator in all models using an effect-coded binary variable. Handedness was included in all MLR models as a nuisance covariate using an effect-coded binary variable. A model selection process was used to find the best-fitting MLR model at each functional ROI, defined as the most parsimonious model that explains a significant amount of between-person variance (per model *F*-test). This iterative process began with a 3-way interaction model (e.g., Group × AUDIT Problem × Sex) and involved dropping non-significant interaction effects followed by non-significant main effects. The threshold for significance was *p* < 0.05. If the process arrived at a best-fitting MLR model containing a significant main or interaction effect of Group or one of its potential moderators, then the robustness of that MLR model to exclusion of statistical outliers or highly influential datapoints was examined. Only MLR models for which the model *F*-test and the relevant beta coefficient *t*-test remained significant after exclusion of such datapoints were considered sufficiently robust to report. Decomposition of significant effects in reported MLR models involved pairwise comparisons of simple slopes or model-estimated means. Bonferroni correction for multiple comparisons was applied to control the Type 1 inferential error rate.

## 3 Results

### 3.1 Behavior

**FIGURE 1:**
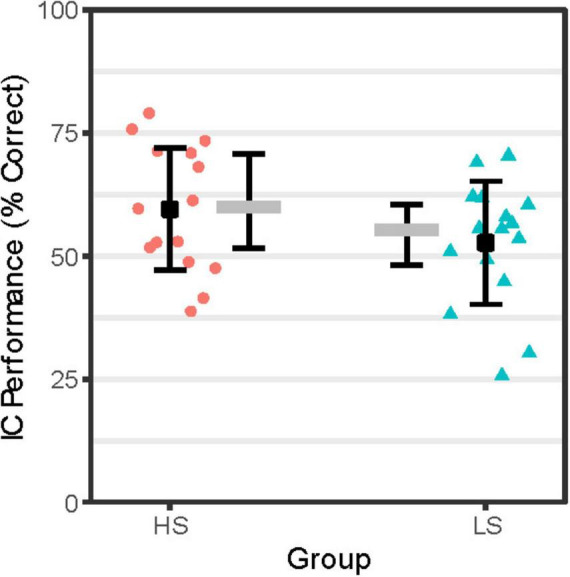
IC Performance by Group. IC performance (% correct = proportion correct × 100) reflects accuracy on NoGo trials adjusted for attentional lapses by scoring NoGo trial null responses coded as incorrect if response to immediately prior FreqGo trial was omitted. Person-level IC performance scores for the High Alcohol Sensitivity (*n* = 16; HS) and Low Alcohol Sensitivity (*n* = 16; LS) groups are shown as red-filled circles and teal-filled triangles, respectively. Group-level mean IC performance scores are shown as black-filled squares flanked by error bars representing ± 1 SE. Group-level median IC performance scores are shown as gray-filled rectangles flanked by error bars indicating the interquartile range.

As shown in Figure 1, IC performance adjusted for attentional lapses was numerically lower for group LS compared to HS. However, as shown in Table 1, no significant group differences were detected on IC performance or other aspects of task behavior (e.g., Go or Rare Go correct RT, premature response counts).

**TABLE 1:** IC task behavior by group.

	HS	LS	Group difference
**Accuracy (proportion correct)**			
	**M (SD)**	**M (SD)**	***T*, *p***
Frequent Go	0.980 (0.019)	0.973 (0.029)	0.83, 0.412
Rare Go	0.973 (0.027)	0.967 (0.035)	0.52, 0.607
NoGo	0.604 (0.124)	0.540 (0.137)	1.37, 0.180
Adjusted NoGo	0.595 (0.124)	0.527 (0.125)	1.55, 0.132
**Premature responses–count**			
	**Med (IQR)**	**Med (IQR)**	***U*,** ***p***
Frequent Go	31 (45.5)	23.5 (28)	137, 0.734
Rare Go	2.5 (5.25)	3.5 (5.50)	117, 0.689
**Omitted responses–count**			
	**Med (IQR)**	**Med (IQR)**	***U*, *p***
Frequent Go	6.5 (12.75)	6.5 (15.00)	108, 0.472
Rare Go	1.00 (2.25)	2.00 (3.00)	116, 0.671
**Response time (milliseconds)**			
	**Med (IQR)**	**Med (IQR)**	***U*, *p***
All correct Frequent Go responses	313.43 (60.89)	319.43 (38.27)	131, 0.926
All correct Rare Go responses	391.35 (65.77)	378.06 (61.52)	158, 0.270
All NoGo responses (commission errors)	290.61 (44.03)	292.60 (31.26)	114, 0.616

HS, High Alcohol Sensitivity; LS, Low Alcohol Sensitivity. *N* = 32 (16 HS, 16 LS, 9 females/group). Adjusted NoGo (primary IC performance index) = NoGo proportion correct adjusted for lapses in attention (e.g., omission not counted as correct if also omitted response to the Go trial immediately prior to the NoGo trial). Premature responses were those with response time (RT) faster than 200 milliseconds. Group comparisons on accuracy measures used the two-tailed independent samples Student’s *t*-test whereas the Wilcoxon rank sum test was used for other measures (e.g., counts, RTs). *t*-test results for accuracy did not change when using angularized (transformed) scores, so raw scores and *t*-test results are presented for ease of comparison with prior reports. Wilcoxon rank sum tests on accuracy scores also indicated no significant difference in median accuracy by group.

### 3.2 Brain

As shown in Table 2, the whole-brain 2nd level model of successful IC-related activity detected 8 clusters spanning cortex, striatum, and cerebellum. Cortical clusters were located primarily in anterior cingulate. Subcortical clusters were located primarily in rostral dorsal striatum (caudate and putamen). Among these functional ROIs, only two located in the dorsal/supragenual anterior cingulate cortex (dACC) exhibited robust effects of alcohol sensitivity phenotype (Group). These Group effects were moderated by biological sex and between-person differences in alcohol craving frequency/strength outside the lab. We present and decompose these Group effects below.

**TABLE 2:** Regions of successful IC-related activity identified using whole-brain 2nd level model across full sample.

Cluster	Cluster size (# voxels)	Activation volume (mm^3^)	MNI coordinates (X, Y, Z) for peak voxel	Anatomical area of peak voxel
1	1,260	4,252.5	27, 20, −4	Putamen_R
			10, 12, −1	Caudate_R
			16, 11, −7	Putamen_R
2	316	1,066.5	−18, 8, −4	Putamen_L
			−26, 11, −2	Putamen_L
			−28, 18, −4	Insula_L
3	98	330.75	9, 34, 22	Cingulum_Ant_R
			2, 36, 26	Cingulum_Ant_R
4	9	30.375	−36, 14, 2	Insula_L
5	3	10.125	−8, 34, 18	Cingulum_Ant_L
6	15	50.625	−32, −54, −30	Cerebelum_6_L
7	4	13.5	−4, 34, 20	Cingulum_Ant_L
8	3	10.125	−4, 38, 11	Cingulum_Ant_L

Successful IC-related activity = IC contrast (NoGo_correct_ BOLD—RareGo_correct_ BOLD). MNI, Montreal Neurological Institute. In the anatomical area of peak voxel column, the specific area label taken from the automated anatomical labeling (AAL) atlas is presented. Activation volume = cluster size (# voxels) × voxel size (1.5 mm^3^). In cases with multiple peak voxels per cluster, peak voxels were significantly different from each other at *p*_FWE_ < 0.05. Full sample *N* = 32.

#### 3.2.1 Left dorsal/supragenual ACC (L-dACC)

##### 3.2.1.1 Group × craving

**FIGURE 2:**
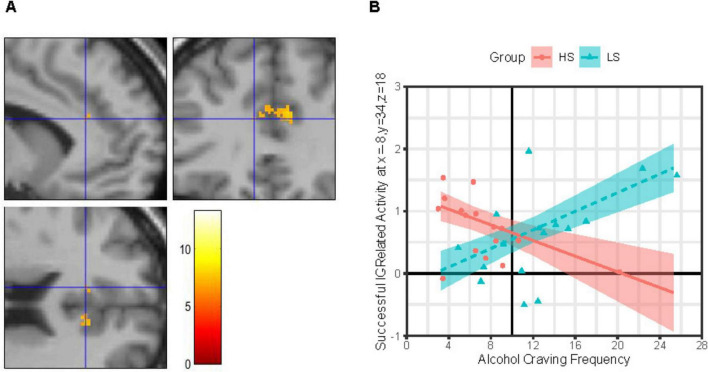
Group × alcohol craving frequency effects on IC-related activity in left dorsal anterior cingulate. **(Panel A)** Images (scale: 80 mm × 80 mm; clockwise: sagittal, coronal, axial views) showing clusters of significant IC contrast (NoGo_correct_ BOLD—RareGo_correct_ BOLD) in the dorsal anterior cingulate cortex (dACC) across the full sample (*N* = 32). Blue crosshair in each image shows the approximate location of an IC-related activity peak at voxel x = –8, y = 34, z = 18 in the left dACC. Color bar shows T-scores. **(Panel B)** Successful IC-related activity = IC contrast. Alcohol Craving Frequency = ACEQ-F subscale scores. Person-level average IC contrast beta coefficients across a 5-mm radius sphere centered on voxel x = –8, y = 34, z = 18 are shown for the High Alcohol Sensitivity (*n* = 16; HS) and Low Alcohol Sensitivity (*n* = 16; LS) groups are shown as red-filled circles and teal-filled triangles, respectively. Multiple linear regression (MLR) model predicted IC activity values (means) across ACEQ-F scores are shown for group HS and LS as a red solid line and dashed teal line, respectively, with color-matched areas around them showing ± 1 SE. Results were robust to removal of statistical outliers. ACEQ-F score was entered into the MLR model as a grand-mean centered predictor. The grand-mean ACEQ-F score is shown as a solid black vertical line intersecting the *x*-axis.

IC-related activity at a site in the L-dACC (Figure 2A) was best explained by a MLR model of Group × ACEQ-F interaction, model *F*(4, 27) = 3.23, *p* = 0.027, *adj. R*^2^ = 0.22, improvements in model fit relative to a main effects only model: Δ model *F* = 10.76, Δ residual *df* = 1, *p* = 0.003, Δ *adj. R*^2^ = 0.27. As shown in Figure 2B, follow-up tests revealed that this L-dACC activity was associated with the *frequency* of daily life alcohol craving experiences, albeit in different directions depending on alcohol sensitivity. Specifically, for group LS, L-dACC activity during IC increased as the frequency of alcohol craving experiences increased; this tendency was statistically robust (simple slope b ± SE = 0.075 ± 0.026, 95% CI: [0.022, 0.128], *p* = 0.007, Bonferroni-corrected *p* = 0.014). In contrast, for group HS, L-dACC activity during IC decreased as the frequency of alcohol craving experiences increased, but this tendency was not statistically robust (simple slope b ± SE = −0.063 ± 0.033, 95% CI: [−0.131, 0.005], *p* = 0.068, Bonferroni-corrected *p* = 0.136).

#### 3.2.2 Right dorsal/supragenual ACC (R-dACC)

**FIGURE 3:**
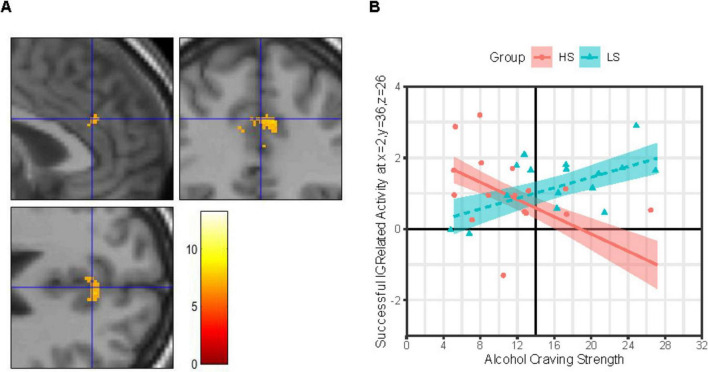
Group × alcohol craving strength effects on IC-related activity in right dorsal anterior cingulate. **(Panel A)** Images (scale: 80 mm × 80 mm; clockwise: sagittal, coronal, axial views) showing clusters of significant IC contrast (NoGo_correct_ BOLD—RareGo_correct_ BOLD) in the dorsal anterior cingulate cortex (dACC) across the full sample (*N* = 32). Blue crosshair in each image shows the approximate location of an IC-related activity peak at voxel x = 2, y = 36, z = 26 in right dACC. Color bar shows T-scores. **(Panel B)** Successful IC-related activity = IC contrast. Alcohol Craving Strength = ACEQ-S subscale scores. Person-level average IC contrast beta coefficients across a 5-mm radius sphere centered on voxel x = 2, y = 36, z = 26 are shown for the High Alcohol Sensitivity (*n* = 16; HS) and Low Alcohol Sensitivity (*n* = 16; LS) groups are shown as red-filled circles and teal-filled triangles, respectively. Multiple linear regression (MLR) model predicted IC activity values (means) across ACEQ-S scores are shown for group HS and LS as a red solid line and dashed teal line, respectively, with color-matched areas around them showing ± 1 SE. Results were robust to removal of statistical outliers. ACEQ-S score was entered into the MLR model as a grand-mean centered predictor. The grand-mean ACEQ-S score is shown as a solid black vertical line intersecting the *x*-axis.

IC-related activity at a site in the R-dACC (Figure 3A) was best explained by a MLR model including both a Group × ACEQ-S and a Group × Sex interaction, model *F*(6, 25) = 3.71, *p* = 0.009, *adj. R*^2^ = 0.34, improvements in model fit relative to a main effects only model: Δ model *F* = 8.51, Δ residual *df* = 2, *p* = 0.001, Δ *adj. R*^2^ = 0.36. Both the Group × Sex interaction, b ± SE = 0.332 ± 0.143, *t*(25) = 2.32, *p* = 0.029, and Group × ACEQ-S interaction, b ± SE = 0.098 ± 0.025, *t*(25) = 3.91, *p* = 0.001, were significant. Follow-up on each is presented next.

##### 3.2.2.1 Group × craving

As shown in Figure 3B, IC-related activity at this R-dACC site was associated with the *strength* of daily life alcohol craving experiences, albeit in different directions depending on alcohol sensitivity. Specifically, for group LS, this R-dACC activity increased as the strength of alcohol craving experiences increased, but this tendency was not statistically robust (simple slope b ± SE = 0.074 ± 0.032, 95% CI: [0.009, 0.140], *p* = 0.028, Bonferroni-corrected *p* = 0.056). In contrast, for group HS, R-dACC activity decreased as the strength of alcohol craving experiences increased, and this tendency was statistically robust (simple slope b ± SE = −0.121 ± 0.039, 95% CI: [−0.201, −0.041], *p* = 0.004, Bonferroni-corrected *p* = 0.009).

##### 3.2.2.2 Group × sex

There was significantly less IC-related activity at this R-dACC site for group HS males compared to group HS females (M_D_ ± SE_D_ = 1.40 ± 0.45, *t*[25] = 3.12, *p* = 0.004, Bonferroni-corrected *p* = 0.018). Additionally, this R-dACC activity was numerically lower for males in group HS than LS, but the difference was not statistically robust (M_D_ ± SE_D_ = 1.08 ± 0.46, *t*[25] = 2.33, *p* = 0.028, Bonferroni-corrected *p* = 0.112). In contrast, no sex difference in R-dACC activity level was detected in group LS (M_D_ ± SE_D_ = 0.68 ± 0.39, *t*[25] = 0.17, *p* = 0.863, Bonferroni-corrected *p* = 1), and R-dACC activity levels were similar for females in group HS compared to group LS (M_D_ ± SE_D_ = 0.24 ± 0.37, *t*[25] = 0.655, *p* = 0.519, Bonferroni-corrected *p* = 1).

## 4 Discussion

The present study found that groups LS and HS were similarly successful at IC task performance, and that at most “hotspots” of IC-related activity detected across the whole brain in the full sample, which implicated the indirect rather than hyperdirect IC pathway, groups LS and HS exhibited similar levels of IC-related activity. There were only two exceptions to the latter finding, both dependent upon accounting for lived experiences of alcohol craving, which can contribute to AUD symptomatology. First, the *frequency* of alcohol cravings experienced outside the lab was associated with IC-related activity at a site in the left anterior cingulate; this association was positive for group LS but negative for group HS. Second, and similarly, as the *strength* of alcohol cravings experienced outside the lab increased, IC-related activity at a site in the right anterior cingulate tended to increase for group LS but to decrease for group HS. The similarity of these association patterns in the dACC across both hemispheres, and across frequency vs. strength dimensions of alcohol craving, suggests a more general link between alcohol craving and anterior cingulate activity, and that this more general link may differ by alcohol sensitivity phenotype. There also appeared to be a sex difference (females > males) in the level of IC-related activity in the right dACC for group HS but not LS. Together, the present findings indicate that, despite a similar capacity to exert control over prepotent responses in the sober state, there may be covert neurofunctional differences in the implementation of IC as a function of alcohol sensitivity phenotype and potential nuances as a function of biological sex.

The present study replicates and extends the one prior fMRI study (to our knowledge) that reported on domain-general IC performance and its neural substrates among LS and HS young adults (Schuckit et al., 2012). Findings from that study indicated similar IC task performance between LS and HS groups but higher activity in left superior frontal gyrus and anterior cingulate during successful IC in group LS compared to group HS. Despite different methods across studies (e.g., recruitment and screening, alcohol sensitivity assessment, IC task, MRI scanner and head coil, and fMRI acquisition parameters), the present study’s findings converge with those reported by Schuckit et al. (2012) in suggesting differential recruitment of, or activation thresholds for, the frontocortical neural substrates of IC in the sober state as a function of alcohol sensitivity phenotype. Although the extent of overlap is unclear, both studies detected this difference at a site in the left anterior cingulate. Furthermore, the present study detected a potential group difference at a site in the right anterior cingulate. This convergence of potential differences being localized bilaterally to anterior cingulate is consistent with the anterior cingulate’s proposed role in IC, which is based on its ability to detect conflict and broadcast cognitive control demand (Botvinick et al., 2004; Shenhav et al., 2016).

More broadly, our convergent fMRI findings with respect to the IC facet of EF reinforce Schuckit et al.’ proposal (2012) that, to perform EF tasks while sober at the same level as HS peers, LS individuals may “over-recruit” the frontocortical nodes of the relevant neural circuits. Additional support for this proposal comes from Schuckit and colleagues’ prior fMRI studies of a different facet of EF: working memory. These studies uncovered greater working memory load-related activation of frontocortical nodes such as anterior cingulate, dorsolateral prefrontal cortex, and inferior frontal gyrus (ventrolateral prefrontal cortex) in the sober state among LS youth compared to HS peers despite similar task performance (Paulus et al., 2006; Tapert et al., 2004; Trim et al., 2010). These sober-state differences between LS and HS individuals in task-related frontocortical activation may even extend from the cognitive to affective task domain (Paulus et al., 2012).

### 4.1 Implications for prevention and treatment of AUD

The present study’s findings have implications for prevention and treatment of AUD. Specifically, the findings suggest that emerging adults reporting LS to alcohol may expend more neural effort or resources to exert control over prepotent responses (in general) than do their peers reporting HS. Prior studies suggest that, compared to their HS peers, LS individuals exhibit amplified approach responses to alcohol cues (Cofresí et al., 2022a; Fleming and Bartholow, 2014) and increased internal conflict or difficulty with IC in alcohol cue-saturated contexts (Bailey and Bartholow, 2016; Cofresí et al., 2022a; Fleming and Bartholow, 2014). Thus, LS persons may be at a disadvantage in the sustainability of their intentions to abstain or moderate alcohol use via deliberate acts of self-control in-the-moment. Supporting abstinence or moderation goals in clients or patients reporting LS to alcohol could involve fostering skills or strategies that minimize the need for deliberate acts of control, or training countervailing prepotent responses to personally relevant alcohol cues. Alternatively, it could involve repeated non-invasive stimulation of the neural circuits supporting self-control, which has been shown to have short- and long-term benefits in AUD and other SUDs (Mehta et al., 2024; Song et al., 2019; Upton et al., 2023a; Upton et al., 2023b). With respect to the latter, the present study suggests that LS individuals may need higher doses or longer courses of treatment than HS peers. Additionally, the present study suggests that LS individuals may derive more benefit from stimulation of the anterior cingulate than from stimulation of other frontocortical sites implicated in self-control that are more common treatment targets, such as the dorsolateral prefrontal cortex (Vanderhasselt et al., 2020; Wietschorke et al., 2016). Alcohol sensitivity phenotype differences also could help explain some of the inconsistency in treatment response thought to underlie inconsistent clinical trial results for non-invasive stimulation of the more common treatment targets (den Uyl et al., 2017, 2018). Non-invasive stimulation targeting the anterior cingulate may bolster LS individuals’ ability to inhibit attentional, approach, or craving responses to alcohol cues, as it has been shown to do generally in certain clinical trials (De Ridder et al., 2011; Kearney-Ramos et al., 2018), potentially by decreasing functional connectivity between anterior cingulate and dorsal striatum (Harel et al., 2022), both of which were found to be activated during successful IC in the present study.

### 4.2 Limitations

The present study’s findings need to be considered in light of the study’s limitations. First and foremost, this study was designed as a preliminary exploration, and therefore its sample size was small. However, individuals with relatively extreme LS and HS phenotypes were recruited to capitalize on phenotypic differences in neurocognitive processes of interest, and yet, the resulting groups also differed on aspects of alcohol craving, consumption, and consequences. The analytic approach provided an efficient means by which to test alcohol sensitivity phenotype-based differences across sites in the brain exhibiting significant IC-related activation while statistically accounting for phenotype-linked covariates indexing AUD risk, such as craving, consumption, and consequences. Nonetheless, future studies seeking to isolate alcohol sensitivity phenotype effects from chronic alcohol use effects (Karoly et al., 2024; Pérez-García et al., 2022) would benefit from stratifying the study sample for alcohol sensitivity and alcohol use levels. Larger studies are necessary to obtain uniform representation across the alcohol use and sensitivity phenotype spectra. As a proposed AUD risk-conferring endophenotype, it is also important to determine which neurocognitive or neurofunctional vulnerabilities associated with LS to alcohol are causes or consequences of alcohol use. Cross-sectional observations like the present study cannot speak to the cause/consequence conundrum. Longitudinal observations are necessary to parse cause from consequence. Furthermore, future studies should consider the extent to which LS to alcohol is associated with specific neuroanatomical variations (e.g., gray matter density in frontal cortices, integrity of white matter tracts linking cortical regions or subcortical nuclei) and their overlap with neuroanatomical variations associated with problematic alcohol use (for review, see Honarvar et al., 2023).

Future studies also should assess other proposed and often related AUD risk-conferring phenotypes (e.g., positive family history, trait disinhibition/impulsivity) and disentangle their contributions from those of alcohol sensitivity phenotype. This is especially important with respect to positive family history (FHP) of AUD because: (i) LS to alcohol was first proposed as a sub-mechanism of FHP-based risk for AUD (Eng et al., 2005; Schuckit, 1980), and (ii) LS to alcohol is a highly heritable source of risk for AUD (Heath et al., 1999; Heath and Martin, 1991; Viken et al., 2003). Prospective studies of AUD onset in large samples indicate that FHP and LS operate as distinct contributors of risk for AUD (Schuckit et al., 2006; Schuckit and Smith, 2000, 2001), which suggests distinct genetic bases. Nonetheless, to the extent that FHP-based risk for AUD and LS-based risk for AUD share common genetic bases (Schuckit, 2018), the present study’s findings also converge with prior fMRI studies of IC as a function of family history of AUD. These prior studies found elevated IC-related activity in anterior cingulate as well as middle and inferior frontal gyri among FHP individuals compared to peers with no family history of AUD [(Heitzeg et al., 2010; Jamadar et al., 2012; Kareken et al., 2013), but also see (Schweinsburg et al., 2004)]. Given that these and other important constructs are being assessed alongside structural and functional neuroimaging repeatedly across development for a nationally representative and extremely large sample [e.g., the NIH Adolescent Brain Cognitive Development (ABCD) Study (Brown S. A. et al., 2023)], a more comprehensive picture of the inter-relatedness or uniqueness of endophenotypic risk factors for AUD onset may soon emerge. These large-scale neuroimaging studies also stand to illuminate the inter-relatedness or uniqueness of AUD risk conferred by neurofunctional (Morales et al., 2024) vs. neuroanatomical characteristics across development (Honarvar et al., 2023; Jones et al., 2023; Miller et al., 2024).

Finally, the present study involved healthy, predominantly White, highly educated participants (university students, presumably from higher socioeconomic status backgrounds) in either late adolescence or early emerging adulthood, which limits generalizability of its findings regarding IC performance and its neural substrates. This limitation also applies to prior fMRI studies of alcohol sensitivity phenotype-based group differences in mental functions (Paulus et al., 2006, 2012; Schuckit et al., 2012; Tapert et al., 2004; Trim et al., 2010). Data from an extremely large and nationally representative sample like the ABCD Study can be leveraged to examine measurement invariance across ethnic, racial, and socioeconomic status groups for alcohol sensitivity and AUD risk as well as for IC performance and its neural substrates.

## 5 Conclusion

Preliminary findings from the present fMRI pilot study suggest that the ability to implement IC over prepotent responses to cues (broadly) among individuals with LS to alcohol may depend on compensatory over-activation of the anterior cingulate cortex, and that this compensatory activation tracks dimensions of their alcohol craving experiences in daily life. Prior fMRI studies of IC as a function of LS to alcohol or positive family history of AUD using different samples, assessments, and tasks also point to compensatory over-activation of anterior cingulate cortex or functionally related frontocortical regions. Longitudinal fMRI studies with larger and more demographically diverse samples are needed to examine the extent to which covert functional differences in the neural substrates of IC can account for differential AUD onset or progression risk as a function of alcohol sensitivity phenotype.

## Data Availability

The raw data supporting the conclusions of this article will be made available by the authors, without undue reservation.
