# Supplementary material for: Inhibitory control in the sober state as a function of alcohol sensitivity: a pilot functional magnetic resonance imaging (fMRI) study
Source: Front Hum Neurosci. 2025 Feb 28;19:1557661. doi: 10.3389/fnhum.2025.1557661 (PMC11906719; doi:10.3389/fnhum.2025.1557661)
Supplement: Supplementary file 1 [file Table_1.docx]

| **Table S1**  *AUDIT and ACEQ subscale scores by Group* | | | |
| --- | --- | --- | --- |
|  | HS | LS | Group Difference |
|  | M (SD) | M (SD) | *T, p* |
| AUDIT-C | 4.31 (1.74) | 7.75 (1.81) | 5.48, <.001 |
| AUDIT-P | 2.50 (3.01) | 8.00 (5.73) | 3.40, .002 |
| ACEQ-F | 6.94 (4.19) | 12.44 (5.37) | 3.23, .003 |
| ACEQ-S | 11.19 (5.52) | 16.50 (6.20) | 2.56, .016 |
| *Note*. AUDIT = Alcohol Use Disorders Identification Test. ACEQ = Alcohol Craving Experience Questionnaire. AUDIT-C = AUDIT Consumption subscale. AUDIT-P = AUDIT Problems subscale. ACEQ-F = ACEQ Frequency form, Intensity subscale. ACEQ-S = ACEQ Strength form, Intensity subscale. HS = High Alcohol Sensitivity Group (n=16 [9 females]). LS = Low Alcohol Sensitivity Group (n=16 [9 females]). Group comparisons used the two-tailed independent samples Student’s *T*-test. Welch’s *T*-test, which adjusts for unequal variance across groups, returned similar results, as did Wilcoxon rank sum tests. | | | |
