# Supplementary material for: Inhibitory control in the sober state as a function of alcohol sensitivity: a pilot functional magnetic resonance imaging (fMRI) study
Source: Front Hum Neurosci. 2025 Feb 28;19:1557661. doi: 10.3389/fnhum.2025.1557661 (PMC11906719; doi:10.3389/fnhum.2025.1557661)
Supplement: Supplementary file 2 [file Table_2.docx]

| **Table S2**  *AUDIT and ACEQ subscale score correlation matrix collapsing Group* | | | | |
| --- | --- | --- | --- | --- |
|  | AUDIT-C | AUDIT-P | ACEQ-F | ACEQ-S |
| AUDIT-C | - | - | - | - |
| AUDIT-P | .76*** | - | - | - |
| ACEQ-F | .62*** | .59*** | - | - |
| ACEQ-S | .44* | .40* | .43* | - |
| *Note*. Pearson correlation coefficients shown. Results were similar for Spearman (rank) correlation coefficient tests. AUDIT = Alcohol Use Disorders Identification Test. ACEQ = Alcohol Craving Experience Questionnaire. AUDIT-C = AUDIT Consumption subscale. AUDIT-P = AUDIT Problems subscale. ACEQ-F = ACEQ Frequency form, Intensity subscale. ACEQ-S = ACEQ Strength form, Intensity subscale. Total *N* = 32 (n=16 per Alcohol Sensitivity Group [9 females/group]).  * = *p*<.05  ** = *p*<.01  *** = *p*<.001 | | | | |
